# A Qualitative Study of Barriers and Facilitators to the Uptake of Cardiac Rehabilitation in Octogenarians

**DOI:** 10.3390/geriatrics9060161

**Published:** 2024-12-13

**Authors:** Charlotte Nichol, Rajiv Das, Gill Barry, Michael Kelly, Ioannis Vogiatzis, Nicola Adams

**Affiliations:** 1 Department of Sport, Exercise and Rehabilitation, Faculty of Health and Life Sciences, Northumbria University, Newcastle-upon-Tyne NE1 8ST, UK; charlotte2.nichol@northumbria.ac.uk (C.N.); rajiv.das1@nhs.net (R.D.); gill.barry@northumbria.ac.uk (G.B.); michael4.kelly@northumbria.ac.uk (M.K.); ioannis.vogiatzis@northumbria.ac.uk (I.V.); 2 Cardiothoracic Centre, Freeman Hospital, Newcastle-upon-Tyne NHS Hospitals NHS Foundation Trust, Tyne and Wear NE7 7DN, UK

**Keywords:** cardiac rehabilitation, qualitative, older patients’ perspectives

## Abstract

**Introduction:** Despite an established evidence-base for cardiac rehabilitation (CR) improving functional outcomes and quality of life and reducing re-hospitalisation, there is limited research on CR for older cardiac patients, who require rehabilitation the most, as they are often very deconditioned due to aortic stenosis (AS). CR uptake in the UK is limited to 52% with national variability of provision and accessibility, and it is a national priority to increase uptake to 85%. Frequently, research has excluded older populations as they are deemed to be too frail or generally not suitable for inclusion. This study aimed to explore factors that can impact the uptake of CR in octogenarians. **Methods:** Qualitative interviews were carried out with 20 AS patients (12 female, 8 male), from a large NHS Trust in the North East of England. **Results:** Four main themes were identified in the data: Perceptions and Understanding, Delivery and Accessibility, Perceived Impact of Exercise and Health and Life Changes, and Transportation. **Discussion:** The findings suggested that the major factors were the understanding of the nature, purpose and relevance of CR to older patients, whether CR was offered, and the role of social support. Barriers and facilitators can impact uptake based on the mode of delivery and the individual circumstances identified. Future research could explore how to develop CR programmes that overcome the barriers identified in the research, such as education, monitoring strategies, use of telehealth, and home-based elements to create an acceptable and accessible programme for octogenarians.

## 1. Introduction

Cardiac Rehabilitation (CR) is often defined as a complex and extensive intervention that is frequently offered to patients following a cardiac surgery or procedure [1,2,3]. CR is a broad concept [4], and it can be difficult to define the precise components. CR generally consists of supervised exercises that patients undertake following their procedure, alongside psychological and lifestyle support, as well as regular evaluations from a medical team [5] with programmes lasting between 6 weeks and 12 months [6]. Uptake for CR is estimated to be 52% in the UK [7], and the NHS Long Term Plan aims for uptake rates to reach a target of 85% by 2028 [8]. However, barriers exist for access and participation, which are especially evident for the older populations [9].

Unreliable or inconvenient transport can limit attendance for patients who are offered CR [4,10,11]. Typically, if patients miss one CR session due to unreliable transport, this can lead to subsequent missed sessions, as non-attendance is easier/preferable than relying on transport that is likely to be cancelled [12], or that may not be available for when they require it. Barriers limiting access to CR programmes can vary from transportation to the location of the programmes, to whether the patient and medical professionals perceive that there is a need for it [13,14]. The previous literature has indicated that if practitioners do not perceive that there is a need for CR following a procedure, they may not actually offer it to the patient [13,14]. A survey by Foster et al., [13], which included 295 CR patients in the Scottish Highlands, indicated that transport was a barrier to attendance, which was also reported in qualitative interviews by Tod et al., [14], which included 15 medical professionals and 20 myocardial infarction patients, based in South Yorkshire, UK.

There is an established evidence base supporting CR for the adult population to improve physical and psychological outcomes. Two Cochrane Systematic Reviews have demonstrated that when patients partake in exercise-based CR, their exercise capacity increases [15] as does their health-related quality of life [3,16]. The existing literature has indicated that taking part in CR can increase functional capacity and quality of life in those who take part [17]. The long-term benefits of CR have indicated patients make healthier lifestyle choices [18] and have more confidence in their ability to engage in more intense exercise [15].

The components that make up CR programmes vary, but there are common components across the programmes. There is a common consensus that exercise plays a key role in rehabilitation [19,20]. Often the goal of CR for the older population is to reduce their frailty and increase their capabilities, and this is frequently carried out by exercising [21]. However, the older population is commonly excluded from the current literature as they are often considered to be too frail or generally not suitable for the research [22,23]. Therefore, it is difficult to determine what the specific benefits are for older patients, and how best to deliver CR for this population.

Older adults are often defined as people over the age of 60 years old [24]. Older cardiac patients were deemed to be the group that would benefit the most from CR following their procedure [25], as they have a higher risk of hospitalisation and comorbidities [26], yet there is, to date, little research that has specifically focused on older patients.

The existing literature does not typically consider the experiences and opinions of the older population regarding their decisions to participate or not in CR if it is offered to them. Few studies that have taken a qualitative approach have suggested that barriers are experienced by patients when it comes to CR and these barriers can include lack of understanding of what CR is, issues with communicating about CR, and the absence of appropriate services [14,27]. However, these studies did not focus solely on older patients but rather on patients between the ages of 37 years old and 82 years old. Although two studies have considered older CR participants, they were conducted in remote areas of the UK [13], which only included some brief responses related to the role of CR or were over 20 years old [14]. The majority of research has been quantitative, focusing on outcome measures, rather than considering patients’ opinions and experiences of CR. Whilst the previous literature does provide an understanding of the CR programmes in a quantifiable way, it often does not provide a deeper understanding of the opinions of patients who may take part in the programmes, and the factors that can have an impact on adherence or uptake. Whilst there is some literature that considers the patients’ opinions and experiences regarding CR, it is not as prevalent in the research especially when considering octogenarians compared to younger components [28].

In the UK for the year 2021–2022, 7601 TAVI procedures were carried out with a mean age of 80.8 years old for patients [29], in the year 2022–2023, 3623 sAVR procedures were carried out [30] and for the same year, 89,660 angioplasty procedures were carried out [31]. A small survey carried out by the British Heart Foundation [32] found that in a sample of 217 cardiac rehabilitation participants, only 16% of those taking part in the programmes were over the age of 75 years, compared to 84% of participants being between the ages of 18 and 74 years old.

The present study aims to explore the factors that impact the uptake of CR in octogenarians, and the perspectives that they have on CR, to provide a more contemporaneous understanding of older patients’ experiences of CR, and what the potential barriers to uptake are. The qualitative findings of the previous literature can be suggested to be limited or outdated when identifying impacting factors for the uptake of CR. The research has proposed three main research questions: ‘What do people know and understand about cardiac rehabilitation?’, ‘How should cardiac rehabilitation be offered to promote the uptake?’, and ‘What can prevent people taking part in cardiac rehabilitation?’.

## 2. Materials and Methods

### 2.1. Epistemology

The current study takes a constructivist approach as it aims to explore perspectives and experiences of patients, rather than exploring the concepts based solely on facts [33]. This approach can indicate that personal experiences can provide a more accurate representation of their perspectives and experiences [34].

### 2.2. Research Approach

A qualitative approach was utilised, using semi-structured interviews. Semi-structured interviews provide the opportunity to gain a more detailed understanding of participants’ views and perspectives [35]. This approach allows for expansion of participants’ responses and can support future research. The Theory of Planned Behaviour [36] informed the development of the interview schedule (Appendix A). The theory suggests that the actions of an individual are controlled by the intentions that they have, and this was deemed to be an appropriate theoretical basis for the present study as it aimed to explore how the intentions of participants could influence their likelihood of taking part in CR (the action). The interview schedule was developed by the research team based on the previous literature and studies as well as the Theory of Planned Behaviour and it is not currently validated. Thematic Analysis was utilised as it would be based on personal understandings and perspectives, and this approach allowed for themes to be easily identified. An inductive approach was taken with the thematic analysis as the research and conclusions were data-driven [37].

### 2.3. Participants

Participants were recruited using purposive sampling. In total, 20 participants were invited to take part in the study (12 female, 8 male), with 18 interviews being undertaken in the final analysis. Participants were identified and screened for eligibility from cardiac clinics overseen by the clinical principal researcher (RD). To be eligible for the study, participants had to be over the age of 80 years old and due to have or have undergone a cardiac procedure for AS. Cardiac procedures included Transcatheter Aortic Valve Implantation (TAVI), Surgical Aortic Valve Replacement (sAVR), or Angioplasty. Participants were recruited regardless of their gender identity, sexual orientation, or previous health conditions. Participants were excluded from the study if they had had any other cardiac procedure or were younger than 80 years old. Recruitment occurred whilst participants were in hospital for a scheduled appointment. In total, 20 participants were selected as this quantity would allow for a variety of themes and ideas to be identified, whilst ensuring the data remained manageable [38]. Participants were provided with a participant number ranging from one to twenty prior to taking part in the study. Numbers were provided to ensure the anonymity of the participants.

### 2.4. Ethical Issues

Ethical approval for the study was obtained from the Health Research Authority NHS Research Ethics Committee (IRAS ID 300018, REC Reference 21/PR/0931, 11 October 2021), prior to the study being carried out. Ethical considerations included obtaining informed consent from participants prior to taking part. Written consent was obtained with participants also receiving a copy of the consent form. Oral consent was received prior to recording the interviews. Participants were informed of their right to withdraw at any point with no impact on their care. Participants were also informed that if they found any of the questions distressing or uncomfortable to answer they did not have to answer them and they were provided with helplines that they could access once the interviews had been completed for additional support.

### 2.5. Data Collection and Procedure

The interviews were carried out in person at a specialist Cardiothoracic Centre in the North East of England, with interviews being conducted between October 2022 and March 2023 by CN.

Following recruitment, participants were provided with an information sheet and a consent form. Participants were provided with an opportunity to ask any questions that they may have had about the study prior to providing consent. Written and verbal consent was obtained prior to participation in the study. Informed consent included a briefing on the interview process and confirmation that participants were free to refrain from answering any questions or withdraw from the study. Interviews were conducted within the hospital ward, with a median duration of 35 min.

The interviews were conducted by a masters-level health psychologist (CN) who has experience conducting qualitative research. In cases where participants did not express any strong opinions related to themselves, they were asked for their opinions in relation to the wider population to attempt to gauge what their responses would be in relation to others. For example, phrasing such as, ‘it might not be applicable to yourself, but do you think in general’ was used to help with the line of questioning. At the end of the interview, all participants briefly reviewed the interviews for verification. The interviews were recorded using a Dictaphone.

Whilst the data collection process was ongoing, the data were checked for emerging themes and were discussed with the wider team who have experience in qualitative research. The study was brought to a close when information power had been reached and no new data was emerging from the interviews.

### 2.6. Analysis

Data were analysed using a thematic approach informed by Braun and Clarke. This analysis was utilised as it allows for themes and ideas to be identified across the dataset to answer the posed research questions. Data were collected and transcribed by the principal researcher (CN). Recordings were reviewed for transcription accuracy. Dara was reviewed by the principal researcher to develop familiarity prior to the analysis. When analysing the data, initial thoughts and comments were noted on the first read through and this indicated the first interactions with the data and the beginning of the coding phase [39]. Initial comments were further developed in the full coding phase and ideas were gathered across the dataset if they were deemed to be relevant to the research questions [40]. Codes were later generated into themes. Codes were grouped into themes based on how similar the data points were. Reviewing the codes was a continuous and reflexive process during the analysis to ensure the codes were appropriately grouped together, and the themes made sense collectively [41]. The analysis phase was a continuous process to ensure that the final groupings were coherent. When themes had been finalised, names and definitions were generated so that they were clear and concise, and contained an appropriate number of codes to allow the theme to be fully analysed. Themes were checked with other members of the research team (NA and MK) and member checking performed. Guided by Shenton (2004) [42] trustworthiness was supported through frequent debriefing sessions, peer scrutiny and reflexive diaries.

## 3. Results

Participant demographics are displayed in Table 1. All participants had either had or were due to have their procedure and were inpatients at the hospital at the time of the interviews.

The analysis identified a range of key themes and concepts that were apparent in the dataset and addressed the main research questions. These themes provide an understanding of what contributes to the uptake of CR in octogenarians and the role that a solid understanding of CR can play. When participants were approached to take part in the study, many expressed that they had not been approached about taking part in CR following their procedure. Four key themes emerged in the data that covered a range of topics related to the uptake of CR to provide elucidation of the issues being addressed. The themes that emerged were: ‘Perceptions and Understanding of Cardiac Rehabilitation’, ‘Delivery and Accessibility of Cardiac Rehabilitation Programmes’, ‘Perceived Impact of Life Factors on Cardiac Rehabilitation and Health’, and ‘Transportation Concerns’ (Appendix B). The data that were provided have clear links across the identified themes and this suggests that the key concepts provided are similar and, therefore, relevant to the research questions.

### 3.1. Perceptions and Understanding of Cardiac Rehabilitation

Perceptions and understanding of CR, and the potential benefits, provided varying responses that impacted decisions to take part. Two sub-themes were identified: ‘Negative Misconceptions and Thoughts’ and ‘Perceived Benefits and the Role of Support’.

#### 3.1.1. Negative Misconceptions and Thoughts

CR was considered to be more suitable for younger populations as they believe they are too old to benefit from it, “I don’t think so not at my age now…leave it to a lot of the middle aged younger people” (Participant 7). Some participants considered that CR was the medical/surgical procedure they had undergone, and they did not need anything further, “The problem has been resolved now so I’m hoping I won’t need rehabilitation” (Participant 2). It was perceived that the procedure itself would bring participants back to a healthy state without the need for additional rehabilitation, “Once I’ve had the operation if I’m going to be hundred percent” (Participant 4).

If participants were physically active before their condition presented itself, they would automatically assume a return to being this active; “I’m hoping that once I’ve no longer got aortic stenosis I’m hoping I can go home…start off slowly and build it up again until I can walk a lot better” (Participant 12). People with less social support or physical capabilities were viewed as needing CR more than the participants as they are not as independent as the participants perceived themselves to be, “A lot of people do need help and that I’m lucky I still have family and my husband a lot of people my age they’re on their own…and they don’t have anyone to turn to” (Participant 5), “It’s got its place um but it’s not the sort of thing I’d find myself doing I I’m a bit independent” (Participant 20).

If there was a lack of information about CR, participants did not feel confident enough to decide to take part, “I’ve had no information whatsoever” (Participant 18). Often medical professionals did not provide the information to participants, and this could lead to the misconceptions that they had; “I couldn’t name them I I couldn’t name them I’ve done I’ve been to a few clinics and things in my time” (Participant 10).

#### 3.1.2. Perceived Benefits and the Role of Support

Participants perceived that CR could be beneficial in that it could improve their physical ability; “I could go back to doing my exercises” (Participant 8). Participants suggested that CR could improve their quality of life and potentially prevent their condition from returning, “Just to have some quality of life that you could feel your quality of life getting there” (Participant 13). Taking part in CR can provide social support and a sense of strength in numbers for the participants as they are surrounded by people in a similar situation to them; “Prepared to talk to somebody about it to share the experience” (Participant 11). Participants did suggest that if they had their own support network, they would be less likely to take part in CR as they had all the additional support they needed without the need for a CR programme, “I’ve got a good family got a good son and daughter and they’re always looking after me” (Participant 14).

### 3.2. Delivery and Accessibility of Cardiac Rehabilitation Programmes

How CR should be delivered varied and was dependent on a participant’s understanding of CR. Hospital settings were often the preferred method as they were perceived to have a more medically professional element to them and a greater perception of safety; “I’ve got to lean heavily towards hospitals they do a fantastic job” (Participant 3). Although hospital settings have a more professional element, home-based settings are considered to be more accessible for participants through the use of virtual means of instruction booklets; “Package of activities in fact I have a heart manual they gave me last time which was very comprehensive eh very well written” (Participant 17), “I’m quite happy if I could receive it at home in one form or another booklet on exercises coming through on the iPad YouTube” (Participant 13).

Some participants expressed reservations about home rehabilitation as they did not feel confident in using technology and could find it too difficult, “Everything’s online and I don’t do online and that really is a big bug bear to me” (Participant 9). Group settings such as community centres could be preferred as they provide an element of social support; “Some people love being in a crowd and doing things together and great encouragement” (Participant 11).

If participants thought they could be embarrassed taking part in CR, they would be less likely to take part, “Fearing of failure fearing of being a drag on the group on feeling if it’s a group thing” (Participant 11), “Any smack of them being embarrassed big, big factor embarrassment” (Participant 20). Participants who discussed this notion indicated that they would not want to be embarrassed in front of a group of people and, therefore, would be less likely to take part in CR if it was delivered in a group setting as they would want to avoid this feeling. Taking part in CR in group settings can provide participants with motivation as it would give them something to do throughout the day; “Something to get out of bed…somewhere to go today” (Participant 9). If participants believed that CR would interfere with their regular routine, they would avoid taking part, “I’m not the sort of person to get actively involved in that sort of thing” (Participant 19).

For CR to be viewed more positively by participants, it should be delivered by a medical professional or expert to make it more credible; “So long as there’s someone in charge who knows what they’re doing” (Participant 5). Education needs to be provided by doctors or healthcare professionals so that participants can understand what CR is and how it would benefit them, “Hospitals give you advice on what’s available” (Participant 8). Participants suggested that information could be delivered through leaflets or online platforms to reach wider audiences; “Advertising putting it put papers in well obviously hospitals” (Participant 18).

### 3.3. Perceived Impact of Life Factors on Cardiac Rehabilitation and Health

The importance of taking part in CR was suggested to be influenced by a person’s lifestyle and individual circumstances. When participants were carers, either currently or previously, they suggested they could not take part in CR as it would take time away from their current, “I’ve been nursing my wife for three years with Alzheimer’s, so it’s rather put a restriction on my outdoor activities” (Participant 20). If participants were to take part, it would take away from the time they would have with their significant other, and this could have a negative impact on their relationships and commitments; “I’ve got such a busy life to take something else n you know with the family and that I mean they call my house nanny’s café cause its always open and free” (Participant 14). Participants indicated that if they had changes to their health, it could prevent them from being confident in carrying out CR activities, “I haven’t got to fall down I haven’t got to slip or I’ll break a bone” (Participant 10). Participants identified that if CR required them to add something extra to their regular schedule, or would impact their health, they would find it difficult to incorporate it into their everyday lives, and this can also be influenced by their understanding of the purpose and relevance of CR to older adults.

### 3.4. Transportation Concerns

Participants had concerns relating to accessing CR, due to transport issues. There was a consensus that transportation was a large barrier for octogenarians. Obtaining reliable transport was a barrier for participants relying on others; “If you haven’t got help with travelling home yes probably it depends if you can drive or if you’ve got a bus pass or if you’re capable of getting there yourself” (Participant 5). Travelling to CR could be considered a hassle if participants have to rely on others for transport, “For me it would be cause I’d have to get a lift cause I would have to have about three buses to three buses and I’ve got my walker” (Participant 16). To facilitate this, the participant expressed that having some form of transport provided would assist them in attending CR, “Where I live if they had like a minibus collected everybody and brought them up for it I mean even if you paid a pound or something like that and then to take you back home erm that would save a lot of hassle getting here” (Participant 16).

Financial concerns can prevent participants from taking part in CR as it can be a burden that they cannot afford, and this was particularly prevalent for participants on a limited budget, “To get a taxi it would be about twenty pound each way I’m only on pension credit” (Participant 16).

If the location was viewed to be inaccessible, it would be considered too difficult to attend, “Most elderly people would find it hard to come here” (Participant 13). Participants expressed that they would not feel confident attending CR if it was late night, “Daytime rather than evening because getting home at night at my age can be difficult” (Participant 9).

## 4. Discussion

### 4.1. Main Findings

A qualitative study was carried out to gain knowledge of patients’ personal perspectives and understandings of cardiac rehabilitation. There were common factors that impacted the uptake of CR in octogenarians. The understanding that participants had of the nature and purpose of CR impacted their perceived need for taking up rehabilitation. If participants had a previous understanding of CR and what CR consisted of, they formed their opinions based on this. If participants did not know much about CR, their perceived need for taking up rehabilitation was based on this lack of understanding. Delivery of CR was often a problem for octogenarians, and their preferred delivery was dependent on how they perceived themselves in terms of needs and abilities and the barriers they identified.

### 4.2. Perceptions and Understanding of Cardiac Rehabilitation

Participants had a varied understanding of what CR was, and this impacted who they viewed would benefit from it. The previous literature indicated that CR is not often offered to older cardiac patients as frequently by health professionals, therefore, older populations make up a smaller percentage of those that are taking part in CR [43]. As older participants did not appear to be regularly offered CR, this may have contributed to their lack of understanding about CR, due to a lack of information regarding it. Specifically, in the North East of England it has previously been suggested that only 33% of cardiac patients were registered to take part in CR [44]. Participants in the present study suggested that they would not benefit from CR and do not fully grasp what it entails as they have frequently been excluded from the research that demonstrates the potential benefits. Often the existing literature has excluded the older populations and specifically octogenarians when researching CR, as often research only includes older adults under the age of 70 years old. It could be suggested that medical professionals may not refer older people to CR due to the lack of research that is carried out on the group and that since patients have not been offered CR, they may not have had the opportunity to decide to participate or not. This reflects the existing literature as it has suggested that older patients often view the medical procedure as the only thing that can be carried out to prevent their cardiac condition from progressing or returning [45], and that can contribute to their misconceptions and misunderstandings.

Findings suggested that if there was social support or a sense of teamwork, it would encourage them to take part and could improve their quality of life and physical abilities as they would not feel alone in their situation. As reported by Woodgate and colleagues [46], patients with higher levels of social support were reported to have higher self-efficacy and physical health-related quality of life compared to those with less support. Social support was found to be related to recovery following a cardiac procedure, but this can vary from patient to patient, and this is in line with what patients have indicated [47]. Similar to what participants have discussed, if families place a level of importance on physical activity, then their support can encourage the uptake of CR [48]. This supports what participants have discussed in the interviews as they have similarly suggested that having some form of social support, whether it be familial or from people in the same situations as them, can be beneficial due to the support and encouragement that they can receive. By drawing upon ‘Social Cognitive Theory’ [49] this could explain how people who have more support, such as the support of others in the same situation as them, would have a higher self-efficacy in terms of carrying out exercises and overcoming barriers to exercises. This mirrors the concepts discussed by some participants as they have expressed that they are likely to take part in CR programmes if they had the support of others around them encouraging them to take part.

### 4.3. Delivery and Accessibility of Cardiac Rehabilitation Programmes

The data demonstrate that participants in the study vary in how they believe CR programmes should be delivered and what factors will impact how accessible they view these programmes to be. There was a perception that CR programmes are often best suited to hospital settings, which may be a reflection that traditionally, 75% of CR have been delivered within hospital settings [42]. CR programmes were considered more acceptable if they were delivered by health professionals, reflecting the existing literature [4]. CR programmes that are delivered by multidisciplinary teams which include doctors who are familiar with the patient’s condition, physiotherapists, specialised nurses, psychologists, and dieticians have been considered to be of high quality and fit with the BACPR Quality Assurance Standards [50,51]. This supports what participants discussed and is something that should be considered when implementing CR programmes to ensure that participants feel adequately supported.

Participants noted that if they were in a group setting with people in a similar position it would be less embarrassing to take part as they would not feel as though they were ‘standing out’ compared to the rest of the group or that they would be holding others back from progressing as well as receiving an element of social support from those in the same position as them. The previous literature suggests that if participants received reassurance from similar people, they were more likely to complete the intervention as they felt reassured about the programme, and any embarrassment decreased over time when supported by the rest of the group [52]. Participants in the present study were able to express that if they did not feel embarrassed taking part, they would prefer for programmes to be carried out in group settings as it would provide them with additional support that they need to aid their recovery. Similarly, hospital or community settings provide a level of ‘camaraderie’ and motivation for completion, which was preferred and considered lacking from home-based programmes [53], which was also expressed by participants.

Home-based CR would be considered by participants, and this has been emerging in the recent literature. Participants suggested that home-based CR allows for flexibility, and this notion was suggested to lead to higher completion rates and allow more people to take part in CR [54]. The existing literature has suggested that home-based CR can allow people to complete the programmes on their own schedule and as often as they feel they need to rather than being limited to a set number of sessions and this is performed through the use of apps, wearable devices and video/telephone conferencing [55]. Often older patients are excluded from this research, so further research needs to be carried out to investigate if this method can be implemented in the older populations.

### 4.4. Perceived Impact of Life Factors on Cardiac Rehabilitation and Health

The understanding a participant has about their lifestyle can impact their perceived need for CR. The role of lifestyle was identified as a factor across participants. One key finding was that commitments to a significant other or family would limit their ability or desire to attend CR programmes. Evidence suggests that when patients are caregivers for their partner, they are less likely to take part in CR as they do not have the support of other family members to facilitate them taking part and this was more prevalent for women [56]. Experiencing comorbidities and changes to health was identified as a barrier to CR uptake in octogenarians. The sample in the current study experienced a high number of comorbidities. The previous literature has also reported that comorbidities such as diabetes negatively affect CR uptake compared to patients without comorbidities [57,58]. The existing literature has suggested that having more comorbidities can lead to decreased referrals to CR programmes [59] and often older adults experience more comorbidities than their younger counterparts, which can suggest why they are either not routinely offered CR or may not take part in programmes when they are offered due to concerns surrounding their comorbidities. Often women in the previous literature have specifically expressed that their comorbidities can prevent them from taking part in CR programmes [60] and this is similar to what was found in the present dataset.

### 4.5. Transportation Concerns

Travel and transport were identified as a barrier for participants. When a person is unable to travel to the location of the programme, they do not take part as it can be too difficult to attend on a regular basis [61]. Older people often rely on others for transport, and when this is missing, they do not tend to take part in CR programmes [10]. This perspective was prevalent in the current study as participants believed CR would be too difficult to attend if they did not have transport or had to rely on others for transport. This was relevant to this geographical region as almost half of the patients lived in a rural location. When the location was perceived to be inaccessible, it would be a barrier to attendance as suggested in the previous literature [17]. Inaccessible locations are identified as venues that are in remote areas or where patients that would attend these programmes live in rural areas [61], and CR can then be inaccessible due to having to travel to more urban locations. If participants believed that they would have to travel to the hospital or to a rehabilitation centre in the city or more urban places, they would be reluctant to attend as they would view this as being inaccessible to them, or too much of a hassle to attend regularly. The views of participants appear to closely mirror what the existing literature has suggested.

### 4.6. Strengths and Limitations

A key strength of this study is that it addresses a gap in the literature by focusing on the views and perspectives of octogenarians, who are frequently excluded from the available literature and have a low uptake of CR. The study has gathered valuable data from a hard-to-reach population to identify barriers and facilitators to aid in improving the uptake of CR. However, the findings have limitations in transferability to the wider older population as the present study was carried out at a single centre in the UK. A further limitation of the study was that only a small sample size was recruited. However, despite this, during the analysis, it was deemed that information power was gained [62], as no new information was emerging from the interviews. Future research could increase sample size, by recruiting patients from different areas of the country to consider regional variations in experience.

### 4.7. Recommendations for Future Research

Future research should consider how to overcome the barriers that have been discussed by the participants of the present study and consider the use of more accessible CR programmes, such as community centre or home-based rehabilitation to identify whether these formats will facilitate octogenarians taking up CR programmes. Future research should utilise technology in formats that are considered ‘acceptable’ by participants such as videos and information booklets that could be utilised to facilitate an at-home or community centre-based programme. As telerehabilitation has been utilised in the previous literature and participants of the present study have expressed openness and willingness to use it for rehabilitation, future research should explore this. Future research should focus upon carrying out studies using telehealth methods such as apps, video conferencing and online videos with this cohort, alongside digital literacy sessions, to identify if it is a feasible option for increasing the uptake of CR in octogenarians.

## 5. Conclusions

The study found that there are many factors that impact the uptake of CR in octogenarians. Participants’ understanding of CR was variable. If participants viewed CR as the procedure that they had undergone, they did not perceive a need for any further rehabilitation. If participants understood what CR was, they believed that they were too old to benefit from it and that was frequently demonstrated in the data of the present study. The findings highlighted that their preference for delivery varied and was based on their understanding of what CR was. Barriers and facilitators were similar across the participants. Future research should consider the factors that were discussed in the present study to develop a suitable and accessible programme for octogenarians.

## Figures and Tables

**Table 1:** Demographics of Participants.

Mean Age	85.15 Years (All Participants) 85.84 Years (Participants over the Age of 80 Years)
Female	12
Male	8
Number of Comorbidities	17
Ischemic Heart Disease	6
Chronic Renal Failure	7
Previous Stroke or TIA	1
Peripheral Vascular Disease	2
Hypertension	8
Atrial Fibrillation	5
Osteoporosis	1
Diabetes	3
Chronic Obstructive Pulmonary Disease	3
Asthma	4
Other Comorbidities	Frailty Pulmonary Fibrosis Angina Lung Cancer Liver Disease Left Ventricular Dysfunction Obesity
Urban Residency	11 Urban 9 Rural

## Data Availability

The datasets presented in this article are not directly available to protect participants’ confidentiality. Requests to access anonymised partial datasets should be addressed to charlotte2.nichol@northumbria.ac.uk.

## Appendix A. Interview Schedule

IRAS ID 300018

Title of Study: Promotion of Uptake of Cardiac Rehabilitation by Octogenarians (Rehabilitat-8)

INTERVIEW SCHEDULE

Background Information

1. Can you tell me a little about yourself and what you enjoy doing?
2. Do you do any physical activity, such as walking or take any other exercise?
3. Did you participate in any physical activity e.g., walking, classes, before your cardiac condition?

Awareness and Views

4. Are you aware of the cardiac rehabilitation that is available? If so, where did you find out about this
5. What do you think about cardiac rehabilitation? Is it something you think would help you?
6. What do you think the benefits might be?
7. Do you think there are any drawbacks?
8. What could we do to promote awareness in local communities?

Information

9. Do you feel you have had enough information about cardiac rehabilitation?
10. What information would you like to have?
11. How would you like to receive this information

Barriers and Facilitators

12. What would make participating in cardiac rehabilitation attractive to you?
13. What would put you off participating?
14. Are there any forms of delivery that might work best for you, for example, you might prefer to go to a community centre in person, or have a package of activities and a weekly phone call at home, or perhaps something you can access online if you have access to a computer?
15. What do you think could be done to help you participate?
16. What do you think could be done to make it enjoyable?

Support

17. What support do you think you would need such as having a designated nurse or physiotherapist, or a contact phone call
18. Any other form of support e.g., travel arrangements, carer

Closing

19. Is there anything else you would like to say that I haven’t asked you.

Many thanks for your time.

## Appendix B. Table of Themes

**Theme 1: Perceptions and Understanding of Cardiac Rehabilitation**	
**Sub-Theme**	**Quotation**
Negative Misconceptions and Thoughts	P7: I don’t think so no not at my age now… I’m getting too old for a lot of things now P14: I’m too old for that for things like that now P8: Hopefully once the medication kicks in I’ll erm be back to normal P19: *** had em a stent put in eh sixteen years ago and fortunately he’s been healthy all since then P12: I’m hoping that once I’ve got no longer got aortic stenosis I’m hoping I can go home… start off slowly and build it up again until I can walk a lot better P20: it’s got its place um but it’s not the sort of thing I’d find myself doing I I’m a but independent P5: probably if they know nothing about it it’s always helpful if you get more information and more help P18: I’m not bothered about it… it’d be good for people who want it
Perceived Benefits and the Role of Support	P8: to keep your mind active as well and your body healthy P9: I can at least do some walking even if I cant to the extent I used to P1: give them something to live for P16: it’ll give you a longer life and quality of life P11: Prepared to talk to somebody about it to share the experience P8: if it’s a group you’ve got the companionship of like other people that are doing it you don’t feel so isolated P13: I think for a lot of people they would perhaps like the sociability wouldn’t they P19: I have very good neighbours if I ever want anything I know I just need to knock or pick the telephone up P14: my neighbours they’re doctors they talk to me and they look after me
Theme 2: Delivery and Accessibility of Cardiac Rehabilitation Programmes	
	Quotation
	P2: probably a hospital setting P7: I’ve learnt a lot since I came into the hospital P12: if it was a leaflet with some diagrams that you could even do in your home I don’t think that would be bad either P17: package of activities in fact I have a heart manual they gave me last time which was very comprehensive eh very well written P2: I don’t use computers or anything P2: not as used to that sort of thing um I mean I do use it I wouldn’t certainly wouldn’t want to use it that much P1: You certainly need people that had that background P3: Nurse come into your home to deliver the rehabilitation…that would be acceptable P11: Conversation with people across the board nutrition eh athletes erm eh medical people P1: Keep up with eh with the new stuff that’s coming on P5: I know you’re educating patients but sometimes doctors need educating P9: if there was a leaflet in the doctors or in the chemist and I could pick it up and ask them how I could get this information P17: extensive posters work in public erm in libraries eh main things for me pool facilities any way any organisation seems to reach out to people public relations P10: I honestly think that television is the best medium every man… and his dog watches it P5: there’s more people there then you think what would be more comfortable with more people with the same thing around them P20: the benefits would be from being involved in a group of people who would who would cause each one of them to lean on each other’s experiences P11: Fearing of failure fearing of being a drag on the group on feeling if it’s a group thing P5: Human contact that’s what they need P8: it would be an outing to look forward to P4: I wouldn’t take on a lot more P13: I’m committed to bridge with partners so I cant change that the activities I do I’m committed and don’t want to lose I don’t want to stop that P14: I’ve got such a busy life to take something else on you know with the family P20: I’ve got medical people in the family P1: what would you achieve at the end of it P9: I would feel as though I was going to do myself some good if there was a leaflet P5: people are just getting this done haven’t been told anything it gives them the chance to talk about it give them some information that could help P18: where would it be first of all for getting there and things like that you know that’s obviously you know what benefit it would do me P9: something that’s not online…I could say can you advise me how I can join that
Theme 3: Perceived Impact of Life Factors on Cardiac Rehabilitation and Health	
	Quotation
	P9: after ***** died and I ended up caring for my parents P10: I lost my husband three years ago at the beginning of lockdown which has limited everything P3: I lost my wife three years ago tragically P7: completely couldn’t walk… people here has told me just to be careful in what I do and how P8: my whole life’s changed in so much as what I’m able to do now P3: I’ve definitely lost my grip P3: once you start and lose grip once you start and slide back little things…bad habit P5: my knees are knackered… I can’t get my breath as much P8: I became blind P8: going from here to the toilet and coming back I’m just breathless P11: being incapacitated one loses confidence P11: dietary restrictions financially there are reasons from stress of work distress when things go wrong
Theme 4: Transportation Concerns	
	Quotation
	P9: I’m quite capable of getting to and from just eh if the bus turns up or you know getting the transport to get there that’s the only thing P19: the only drawback I would have was if I had to travel long distance I mean I would hate to have to travel all the way up to the Freeman P12: it depends where it is I’m I don’t use the buses at the moment and I rely on a friend to take me everywhere and I can’t expect her to run here there and everywhere P18: if they haven’t got travel you know got a car so they need someone to take them P1: subsidised for your eh expenses P10: situation pick up bus on the corner P9: if it was going to add expense to doing the class it would be unnecessary P2: get to here would be become a problem P12: if there was something in the village P16: for me it would be getting up here if they had it in ******* somewhere would be difference P19: I would like to go to our community centre P13: No way I want to go out at night
